# P09 Aztreonam and avibactam synergy for MBL-producing Enterobacterales and patient clinical outcomes in a large UK hospital trust

**DOI:** 10.1093/jacamr/dlaf118.016

**Published:** 2025-07-14

**Authors:** L Ting, S Samin, G Hughes

**Affiliations:** Department of Microbiology (UKHSA), Birmingham Heartlands Hospital, University Hospitals Birmingham, Birmingham, UK; Department of Microbiology (UKHSA), Birmingham Heartlands Hospital, University Hospitals Birmingham, Birmingham, UK; Department of Microbiology (UKHSA), Birmingham Heartlands Hospital, University Hospitals Birmingham, Birmingham, UK; Department of Microbiology, Queen Elizabeth Hospital, University Hospitals Birmingham, Birmingham, UK

## Abstract

**Background:**

Antimicrobial resistance presents a continuously evolving global challenge, with significant resources devoted to treating infections caused by MDR organisms. Certain resistance mechanisms, such as Class B β-lactamases, including NDM pose a particular challenge due to limited treatment options, with associated high morbidity and mortality rates. National UK guidelines recommend that all clinical isolates of carbapenemase-producing Enterobacterales (CPE) be sent to the national reference laboratory (London, UK) for inclusion in the strain archive, with susceptibility testing performed upon request. Following the approval of ceftazidime-avibactam use in the UK in 2022, synergy with aztreonam has been explored as a potential treatment option.

**Objectives:**

Given the increasing incidence of NDM-positive cases at University Hospitals Birmingham (UHB), we investigated the total number of isolates exhibiting this resistance mechanism, the proportion demonstrating synergistic activity between aztreonam and ceftazidime-avibactam, and the clinical outcomes of affected patients.

**Methods:**

Through a retrospective audit, all NDM-positive clinical specimens received at UHB laboratories between August 2022 and August 2024 were identified through the laboratory database. Data on patient demographics (age, gender), specimen type, previous screening results, anti-microbial susceptibility (including avibactam–aztreonam synergy), and clinical outcomes (length of stay, 30 day, 90 day and 1-year mortality) was retrieved from electronic patient records and analysed. NDM-positive isolates from screening samples only and duplicate clinical isolates were excluded from the analysis.

**Results:**

We analysed 73 patients with NDM-positive clinical specimens, of whom 62% (*n*=45) were male, with a median age of 59 (IQR 45-73) years. Positive clinical specimens included urine (*n*=34, 46.6%); blood cultures (*n*=10, 13.7%); respiratory samples (*n*=5, 6.9%); sterile fluids (*n*=5, 6.9%), tissue (*n*=3, 4.1%); bone (*n*=2, 2.7%) and others (*n*=13, 17.8%). In total, 17 (23.3%) patients had NDM-positive clinical specimens after prior NDM-positive screen results. One patient developed an NDM bacteraemia following an NDM-positive urine sample. Of the 73 isolates, 27 were sent to the reference laboratory for additional testing. Within this group, all 27 tested resistant to aztreonam, while 24 tested resistant to ceftazidime-avibactam. Synergy between both antibiotics was observed in all 21 isolates that underwent synergy testing, though the MIC was above 1 mg/L for 4 (19%) of them. The majority (*n*=62, 85%) of clinical specimens were collected during an inpatient episode, with a median length of hospital stay of 24 (IQR 10-51) days. All-cause mortality was observed at 11% (*n*=8) at 30 days, 23% (*n*=17) at 90 days, and 33% (*n*=24) at 1 year.

**Conclusions:**

NDM-positive Enterobacterales infections occur across a broad range of clinical sites, with only a small percentage of patients exhibiting positive screening results before clinical infection. While synergy between ceftazidime-avibactam and aztreonam was observed in all tested isolates, four were still above the MIC, leaving few treatment options. The prolonged hospital stays and high mortality rates emphasize the major public health threat of these pathogens.

